# Repeat Cesarean Section among Pregnant Women in a Tertiary Center of Nepal: A Descriptive Cross-sectional Study

**DOI:** 10.31729/jnma.6597

**Published:** 2022-01-31

**Authors:** Dharma Raj Regmi, Ganesh Dangal, Ashmita Silwal, Dhan Bahadur Shrestha, Prakash Raj Oli, Pravash Budhathoki

**Affiliations:** 1 Department of Obstetrics and Gynecology, Paropakar Maternity and Women's Hospital, Thapathali, Kathmandu, Nepal; 2 Department of Obstetrics and Gynecology, Kathmandu Model Hospital, Kathmandu, Nepal; 3 Bir Hospital Nursing Campus, National Academy of Medical Sciences, Kathmandu, Nepal; 4 Department of Emergency Medicine, Mangalbare Hospital, Morang, Nepal; 5 Department of Internal Medicine, Province Hospital, Surkhet, Nepal; 6 Department of Emergency Medicine, Dr. Iwamura Memorial Hospital, Bhaktapur, Nepal

**Keywords:** *Nepal*, *obstetrics*, *repeat cesarean section*

## Abstract

**Introduction:**

Cesarean Section is the most common obstetrics surgery done for both maternal and fetal indications. There is a rising trend of cesarean section rates which is associated with increased maternal morbidities. This study aims to find out the prevalence of repeat Cesarean Section among women with previous cesarean sections done in a tertiary centre.

**Methods:**

This was a descriptive cross-sectional study conducted in a tertiary care hospital of Nepal from August 2020 to January 2021. Pregnant women with previous Cesarean Section status without other pelvic surgery and medical comorbidities were included and data were collected regarding intraoperative findings. Ethical approval was taken from the Institutional Review Committee (Reference Number: 14). A convenience sampling technique was used. Data were analysed using Statistical Package for the Social Sciences version 22. Point estimate at 95% Confidence Interval was calculated, with frequency and percentage.

**Results:**

Out of 1315 patients undergoing Cesarean Section, the prevalence of Repeat Cesarean Section was found to be 184 (13.99%) (12.11-15.86 at 95% Confidence Interval).

**Conclusions:**

The prevalence of Repeat Cesarean Cection from our study was similar to other studies done in similar settings. Repeat Cesarean Cection confers peri-operative morbidities which adversely affect postoperative recovery. Repeat Cesarean Cection continues to contribute to morbidity over subsequent pregnancies and serious maternal morbidity.

## INTRODUCTION

Cesarean Section (CS) is the most common surgery performed in modern obstetrics and performed for both maternal indications and fetal indications.^1^ The CS rate has increased drastically over the past two decades. A higher rate of CS was associated with a greater risk of maternal and perinatal morbidity and mortality, compared to vaginal delivery.^2,3^

The risk of associated maternal morbidities is increased with repeat CS than fetal morbidities associated with CS, more with higher the number of CS repetitions.^4^ The most common maternal comorbidities associated with Repeat Cesarean section (RCS) are the time of hospitalisation, operating time, dense adhesions, bowel and bladder injury, blood loss and blood transfusion requirements, and need for intensive care, morbid placenta, uterine rupture, and hysterectomy.^5-11^

Studies have shown an increased incidence of maternal morbidities associated with the increased number of CS. This study aims to find out the prevalence of repeat cesarean section among Caesarean sections in a tertiary centre of Nepal.

## METHODS

A descriptive cross-sectional study was conducted in a hospital in Kathmandu, Nepal, from August 2020 to January 2021. The study was approved and vetted by the Institutional Review Committee (Reference Number: 14). All Pregnant ladies with a previous one or more cesarean section with singleton pregnancy at term were included in the study. Women who have medical diseases (like diabetes, hypertension, and heart diseases), with previous pelvic surgery other than CS and those who refuse to give consent were excluded. Convenience sampling was used.

The sample size was calculated by using the formula

n = Z^2^ × (p × q) / e^2^

  = (1.96)^2^ × 0.1436 × (1-0.1436) / (0.02)^2^

  = 1182

Where,

n= sample size
Z= 1.96 at 95% of Confidence Interval
p= prevalence of repeat Cesarean section based on a similar previous study.^12^
q= 1-p
e= margin of error, 2%

The calculated sample size was 1182. After adding a non response rate of 10%, the sample size becomes 1301. However, a total of 1315 women meeting the selection criteria for this study were included in the study. The data was collected using proforma. It included demographic data and details of the medical and obstetric history of women, intraoperative and postoperative maternal morbidities following RCS. The intraoperative observation and review of chart post-operatively regarding possible intraoperative morbidities following RCS: Operative time, difficulty in entering the peritoneal cavity, intra-peritoneal adhesions, scar rupture/dehiscence, an extension of uterine incision/tear, difficult delivery/use of forceps, placenta previa, placenta accreta, uterine atony, excessive blood loss, blood transfusion, hematoma formation, bowel/ureteral/bladder/vessel injuries, hysterectomy, maternal death. The participants were observed in the postoperative ward for three postoperative days and on the 7^th^ postoperative day follow-up for the post-operative morbidities associated with repeat cesarean section: post-operative hemoglobin deficit, postpartum hemorrhage, blood transfusion, paralytic ileus, hematoma, pelvic infection, chest infection, puerperal pyrexia, re-laparotomy, UTI, pulmonary embolism, sepsis, wound infection, wound dehiscence, secondary suturing, ICU admission, length of hospital stay. Data were analysed using Statistical Package for the Social Sciences version 22. Point estimate at 95% Confidence Interval was calculated, with frequency and percentage.

## RESULTS

**Figure 1:**
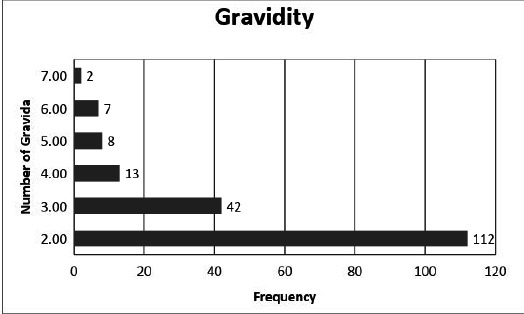
Gravidity status of individuals (n=184).

Out of 1315 patients undergoing Cesarean section, the prevalence of repeat Cesarean section was found to be 184 (13.99%) (12.11-15.86 at 95% Confidence Interval). Among these patients with repeat Cesarean section, most of the cases were in their second gravida followed by third and fourth, and fifth gravida, and 2 cases underwent cesarean section on the seventh gravida (Figure 1).

Among these 184 cases, the majority 149 (81%) were those who had one previous cesarean section, followed by 34 (18.5%) women with previous two cesarean sections and one case with previous three CS. Of all cases, 103 (56%) cases underwent elective cesarean section and the remaining 81 (44%) cases underwent emergency cesarean section. Cephalo-pelvic disproportion was the most common indication for repeat cesarean section followed by fetal distress, oligohydramnios, and so on (Table 1).

**Table 1:** Status, stage of labor, and an indication of repeat cesarean section (n=184).

Variables		n (%)
No of repeat	Previous 1 CS	149 (81.0)
CS	Previous 2 CS	34 (18.5)
	Previous 3 CS	1 (0.5)
Labor trial	Yes	4 (2.2)
	No	180 (97.8)
Stage of labor (SOL)	NIL	116 (63)
	Early SOL	66 (35.9)
before CS	Active SOL	1 (0.5)
	Second SOL	1 (0.5)
Type of CS	Elective	103 (56.0)
	Emergency	81 (44.0)
Indications	Cephalopelvic Disproportion	32 (17.4)
	Fetal Distress	25 (13.6)
	Oligohydramnios	22 (12.0)
	Premature Rupture of Membrane	15 (8.2)
	Intrauterine Growth Restriction	14 (7.6)
	Post Date	13 (7.1)
	Decreased FM	11 (6.0)
	Breech	10 (5.4)
	Scar Tenderness	8 (4.3)
	Placenta Previa	8 (4.3)
	Short Spacing	8 (4.3)
	Unfavourable cervix	8 (4.3)
	Bad Obstetric History	6 (3.3)
	Oblique Lie	3(1.6)
	Hypertension	1 (0.5)

Almost all cases were having longitudinal lie and cephalic presentation and only 11 cases were in breech presentation. In cervical changes, cervical dilation most of the cases were < 1cm, remaining cases were 1-3cm dilated. At the time of repeat cesarean section, the station of presenting part was -2 to -1 in 172 cases at -3 in 10 cases and station 0 or more was found in 2 cases. Among 184 cases, the majority of cases were with intact amniotic membrane and in the remaining cases, the membrane was absent. In repeat cesarean section, the complications may be encountered from the skin incision to the entrance of the peritoneal cavity and then to the lower segment of the uterus. In this study, intraoperative complications were found in 106 (57.6%) cases (Table 2).

**Table 2:** Examination findings and their frequency in repeat cesarean section (n=184).

Variables		n (%)
Lie	Longitudinal	180 (97.8)
	Transverse	1 (0.5)
	Oblique	3(1.6)
Presentation	Cephalic	173 (94.0)
	Breech	11 (6.0)
Cervical dilatation	Less than 1cm	117 (63.6)
	1-3cm	65 (35.3)
	4 or more	2 (1.1)
Cervical effacement	<30%	118 (64.1)
	30-60%	63 (34.2)
	>60%	3(1.6)
Cervical position	Anterior	30 (16.3)
	Mid	143 (77.7)
	Posterior	11 (6.0)
Cervical consistency	Firm	27 (14.7)
	Soft	157 (85.3)
Head/presenting part	-3	10 (5.4)
station	-2 to -1	161 (93.5)
	0 or more	2 (1.1)
Membrane	Membrane present	144 (78.3)
	Membrane absent	40 (21.7)

The presence of adhesions was found among the uterus, rectus muscle, omentum, and in some cases with the urinary bladder too. The uterus having adhesion with rectus muscle and omentum has been seen in an equal number of cases. Adhesion with the urinary bladder was seen in 9 (4.9%) cases only. However, there were no adhesions in 32 (17.4%) cases of adhesion (Table 3). There was difficulty in locating the lower uterine segment in most of the cases. After reaching the lower segment of the uterus, we found that the majority of them had thinned out the lower uterine section, and the remaining cases had a well-formed lower uterine segment. Out of a total of 184 cases, 87 (47.3%) of cases were having thinned out the previous scar, scar dehiscence was present in 52 (28.3%) and the scar was intact in the remaining cases. In this study, we found that the duration of operation was increased with an increase in the number of repeat cesarean sections.

**Table 3:** Intraoperative findings in repeat cesarean section (n = 184).

Variables		n (%)
Adhesion	No	32 (17.4)
	Present with rectus muscle	71 (38.6)
	With omentum	72 (39.1)
	With bladder	9 (4.9)
Lower uterine segment	Well-formed	55 (29.9)
	Thinned out	129 (70.1)
Liquor	Adequate, clear	150 (81.5)
	Adequate, MSL	33 (17.9)
	Minimal, clear	1 (.5)
Placenta	Anterior	22 (12.0)
	Posterior	153 (83.2)
	Low lying	9 (4.9)
Previous scar	Intact	45 (24.5)
	Thinned out	87 (47.3)
	Dehiscence	52 (28.3)
Blood loss	Up to 300ml	27 (14.7)
	>300 and <500ml	134 (72.8)
	500-1000ml	23 (12.5)
Difficulty in delivering head	Yes	99 (53.8)
	No	85 (46.2)
Difficulty in locating	Yes	114 (62.0)
LUS	No	70 (38.0)
Angle extension	Yes	12 (6.5)
	No	172 (93.5)
Uterine atony	Yes	15 (8.2)
	No	169 (91.8)
Bleeding	Yes	22 (12.0)
	No	162 (88.0)
Duration of OT	< 30 minutes	1 (.5)
	30-60 minutes	89 (48.4)
	> 60 minutes	94 (51.1)

Among all participants enrolled in the study, the most common postoperative complication was wound dehiscence followed by post-partum hemorrhage then wound infection that requires secondary suturing. And we found a rare complication, postoperative ileus in 1 (0.5%) case out of 184 women undergoing repeat cesarean section. Among 184 cases, blood was transfused in 9 (4.9%) cases. The most common postoperative deficit of hemoglobin level is < 2g/dl and seen in the majority of cases and only one case, post-operative hemoglobin dropped by > 4g/dl requiring blood transfusion. It is obvious that when the intraoperative or postoperative complications are there then the duration of hospital stay will be increasing. Among 184 cases, most of the cases had a hospital stay of < 5 days and the remaining cases stayed in the hospital for > 5 days. Among all participants, only 147 (79.9%) women came to follow up on the 7^th^ postoperative day for evaluation of any postoperative morbidities, and the remaining 37 (20.1%) did not come for follow-up (Table 4).

**Table 4:** Post-operative complications in repeat cesarean section (n=184).

Variables		n (%)
Wound dehiscence	Yes	26 (14.1)
	No	158 (85.9)
Postpartum	Yes	23 (12.5)
Hemorrhage	No	161 (87.5)
Wound infection	Yes	16 (8.7)
	No	168 (91.3)
Postoperative ileus	Yes	1 (.5)
	No	183 (99.5)
Blood transfusion	Yes	9 (4.9)
	No	175 (95.1)
POP	2 or less	160 (87.0)
Hemoglobin deficit	2-4g/dl	23 (12.5)
	More than 4g/dl	1 (.5)
Inter-delivery spacing	Less than 18 months	9 (4.9)
	18 months - 36 months	53 (28.8)
	More than 36 months	122 (66.3)
Duration of hospital stay	<5 days	139 (75.5)
	5 or more days	45 (24.5)
Follow up on 7^th^ day	Yes	147 (79.9)
	No	37 (20.1)

## DISCUSSION

Cesarean section (CS) is a surgical procedure that may save the life of both the mother and the baby in many cases. In the present study rate of Repeat Cesarean Section was 30% which may be as a result of increased cesarean delivery. While the cesarean birth rate was 4.5% in the USA in 1965, it was 31.8% according to 2007 data and is thought to be over 50% at present.^13-14^ According to the 1993 Turkish Demographic and Health Survey (TDHS), the cesarean birth rate at the time was 8%, and 2008 studies have reported that this has increased to 37%.^15^ There are two significant reasons for this increase: the increasing primary cesarean rate and the rapidly decreasing rate of normal birth after CS, although the final reason for the increase in the primary cesarean rate is not clear, medico-legal issues have probably played an important role.

In our study, the major intraoperative maternal morbidities associated with RCS were the formation of adhesions among the uterus, abdominal wall, and bladder in 82.6%, followed by difficulty in locating the lower uterine segment in 62.0%, prolonged operation time in 51.1%, and previous scar dehiscence in 28.3%. In various studies conducted in different parts of the world had shown that the risk of maternal morbidities associated with Cesarean Section rises with the repeat Cesarean Section and more with higher the parity of the Cesarean Sections.^6-11^ Also, in our study, a significant number of participants had several intraoperative and postoperative morbidities following the repeat cesarean sections.

Almost all participants underwent Repeat Cesarean Section without receiving the trial of the vaginal birth after Cesarean and most of the Cesarean were done before the onset or early stage of the labor. The adhesion among the uterus, abdominal wall, urinary bladder, and other abdominal structures is common intraoperative findings and it may prolong the total operative time duration in repeat Cesarean section cases. In a study conducted by Kaplanoglu, et al. in Southeast Turkey, the rate presence of adhesion among different intra-abdominal structures in RCS case varied from 5.1 to 16.1% depending upon the number of RCS^5^ and that was ranging from 13.5% to 50.0% in a retrospective case-control study done in china by Cintesun, et al.^8^ In this study, the rate of encountering adhesion is quite higher than that in other similar study findings and its 82.6%. The higher rate of the adhesion found in the repeat section could be associated with a higher rate of pelvic inflammatory disease, previous CS section technique, and other undetermined factors. In the same study conducted by Mustafa Kaplanoglu, et al. the operative time for RCS varied from 25.1 to 63.2 minutes, and more time was taken to carry out RCS in women higher number of previous CS.^5^ Similar findings were reported from an observational cohort study by Silver, et al. in Department of Obstetrics and Gynecology, University of Utah School of Medicine USA, 2006 and operative time varied from 50.6 minutes to 79.9 minutes.^10^ In this study, 48.4% of cases had operative time ranging from 30 to 60 minutes while in 51.1% of cases, it took more than 60 minutes.

In this study, the thinned-out LUS is found among 70.1% RCS cases, the difficulty in locating LUS was in 38.0% of cases. In a systematic literature review study by Zwergel, et al. in 2019 , the rate of uterine scar dehiscence was with the rising number of repeat cesarean sections and it varied from 0.43% to 4.34%. In this study, uterine scar dehiscence is found among 28.3% of cases.^11^ In the study, among 12.5 cases the intraoperative blood loss was 500 to 1000 ml, and during the postoperative period, the postpartum hemorrhage was seen among 12.5% of all RCS cases. The blood transfusion was done among 4.9% of RCS cases. The rate of blood transfusion following RCS in this study comparable to that finding from studies by Mustafa Kaplanoglu, et al,^5^ Silver, et al.^10^ Zwergel, et al.^11^

Despite surgical procedures being performed under aseptic conditions, there are no surgical interventions that are free of associated infection of the surgical wound and often its dehiscence. The surgical wound of the RCS is complicated post-operatively by wound infection and wound dehiscence. In this study, the rate of wound infection and wound dehiscences were 8.7% and 14.1% respectively. These rates from this study are more than that in studies by Kaplanoglu, et al.^5^ Silver, et al.^10^ The reason for the higher rate of wound infection and wound dehiscence in this study could be due to an increase in operation time or low protein diet or poor hygiene or longer duration of hospital stay. Nowadays, there is a shift in postoperative care of the patient with a short postoperative hospital stay with early discharge to home due to evolving concepts of the concept of enhanced recovery after surgery. The prolonged hospital stay is considered as one of the postoperative morbidity due to related surgical intervention. In this study, the rate of prolonged hospital stay by 5 days or more is 24.5%. This rate is higher in comparison to that from other similar studies.^5,10^

This study found that the major morbidities associated with maternal outcome during the intraoperative period following the repeat cesarean section are adhesion formation among uterus and surrounding structures, the thinned out LUS with difficulties with locating it, previous scar dehiscence, and the prolonged operation time. While during the postoperative period, the major complications associated are wound dehiscence and wound infection, postpartum hemorrhage and needful of blood transfusion, and the prolonged hospital stay. These major intraoperative and post-operative maternal morbidities following RCS are consistent with the maternal morbidities following RCS shown by other studies. This study failed to observe the association of the morbid placentation associated with previous CS as it is one of the common maternal morbidity found during intra-operative at the time of RCS.

There are some limitations in our study, it was a hospital-based study that was conducted within a short duration of time. We are only able to include 184 women undergoing repeat cesarean section during this 6 months period. Since many of the cases in the study are far from the study place so once they get discharged only very few had follow-up visits in the postoperative period.

## CONCLUSIONS

The prevalence of repeat cesarean section from our study was similar to other studies done in similar settings. Repeat cesarean sections, especially after two cesareans confers peri-operative morbidities adversely affects post-operative recovery. Scar dehiscence and rupture or dense adhesions posing difficult dissection necessitating forceps application and delivery, inadvertently ending in the extension of uterine incision, increased blood loss, need of blood transfusion, prolonged hospital stay corroborates that repeat cesarean section continues to contribute to morbidity over subsequent pregnancies and serious maternal morbidity increases progressively with an increasing number of cesarean deliveries.
